# GWAS for Drought Resilience Traits in Red Clover (*Trifolium pratense* L.)

**DOI:** 10.3390/genes15101347

**Published:** 2024-10-21

**Authors:** Tim Vleugels, Tom Ruttink, Daniel Ariza-Suarez, Reena Dubey, Aamir Saleem, Isabel Roldán-Ruiz, Hilde Muylle

**Affiliations:** 1 Plant Sciences Unit, ILVO (Flanders Research Institute for Agriculture, Fisheries and Food), Caritasstraat 39, 9090 Melle, Belgium; tom.ruttink@ilvo.vlaanderen.be (T.R.);; 2 Department of Plant Biotechnology and Bioinformatics, Faculty of Sciences, Ghent University, Technologiepark 71, 9052 Ghent, Belgium; 3 Molecular Plant Breeding, Institute of Agricultural Sciences, ETH Zurich, Universitaetstrasse 2, 8092 Zurich, Switzerland; 4 Faculty of Bioscience Engineering, Ghent University, Coupure Links 653, 9000 Gent, Belgium; 5 Laboratory of Plant Breeding, Wageningen University & Research, Droevendaalsesteeg 1, 6708 PB Wageningen, The Netherlands

**Keywords:** *Trifolium pratense*, drought responses, GWAS, candidate genes

## Abstract

Red clover (*Trifolium pratense* L.) is a well-appreciated grassland crop in temperate climates but suffers from increasingly frequent and severe drought periods. Molecular markers for drought resilience (DR) would benefit breeding initiatives for red clover, as would a better understanding of the genes involved in DR. Two previous studies, as follows, have: (1) identified phenotypic DR traits in a diverse set of red clover accessions; and (2) produced genotypic data using a pooled genotyping-by-sequencing (GBS) approach in the same collection. In the present study, we performed genome-wide association studies (GWAS) for DR using the available phenotypic and genotypic data. Single nucleotide polymorphism (SNP) calling was performed using GBS data and the following two red clover genome assemblies: the recent HEN-17 assembly and the Milvus assembly. SNP positions with significant associations were used to delineate flanking regions in both genome assemblies, while functional annotations were retrieved from *Medicago truncatula* orthologs. GWAS revealed 19 significant SNPs in the HEN-17-derived SNP set, explaining between 5.3 and 23.2% of the phenotypic variation per SNP–trait combination for DR traits. Among the genes in the SNP-flanking regions, we identified candidate genes related to cell wall structuring, genes encoding sugar-modifying proteins, an ureide permease gene, and other genes linked to stress metabolism pathways. GWAS revealed 29 SNPs in the Milvus-derived SNP set that explained substantially more phenotypic variation for DR traits, between 5.3 and 42.3% per SNP–trait combination. Candidate genes included a DEAD-box ATP-dependent RNA helicase gene, a P-loop nucleoside triphosphate hydrolase gene, a Myb/SANT-like DNA-binding domain protein, and an ubiquitin–protein ligase gene. Most accessions in this study are genetically more closely related to the Milvus genotype than to HEN-17, possibly explaining how the Milvus-derived SNP set yielded more robust associations. The Milvus-derived SNP set pinpointed 10 genomic regions that explained more than 25% of the phenotypic variation for DR traits. A possible next step could be the implementation of these SNP markers in practical breeding programs, which would help to improve DR in red clover. Candidate genes could be further characterized in future research to unravel drought stress resilience in red clover in more detail.

## 1. Introduction

Grasslands cover more than a third of the European agricultural area [1]. They play important roles in livestock feeding and deliver valuable ecosystem services [1]. Red clover (*T. pratense*) is a short-lived perennial legume crop that is often included in grasslands for its nitrogen fixation capacity, its protein-rich forage, and its high palatability [2]. Red clover is naturally a diploid species (2n = 2x = 14) with the following two maturity types: the late flowering single-cut type; and the early flowering, quickly re-growing double-cut type [3]. Red clover is a strictly outcrossing crop, and populations or accessions are genetically and phenotypically highly diverse [4], rendering genetic work generally difficult [5].

Climate change is causing warmer summers with more frequent and more prolonged drought periods in Europe [6]. Grasslands are particularly vulnerable to drought; studies in European grasslands predict annual yield losses between 30% and 40% due to drought [7,8]. The general understanding of plant drought responses and the various coping mechanisms for abiotic stress has advanced significantly in recent years. Comprehensive reviews are available on the physiological basis of drought responses in plants [9] and on drought signaling pathways [10]. Important pathways for drought responses include calcium signaling pathways, MAPK-dependent pathways that control a wide range of developmental and stress–response functions, and pathways involving abscisic acid (ABA) [10]. The phytohormone ABA plays a key role in drought responses, influencing stomatal closure and activating drought-responsive genes through specific pathways [10]. However, other phytohormones, such as salicylic acid, auxin, ethylene, jasmonic acid, brassinosteroid and gibberellin, are involved in drought responses, although their exact roles are often not clear [11]. In addition, transcription factors are crucial components in drought responses as regulators of stress-responsive genes [12].

Red clover is regarded as a rather drought-resilient grassland crop [2], thanks to its deep taproot development [3]. Nonetheless, in the light of climate change, breeding for drought resilience (DR) becomes increasingly important in red clover. However, the cross-pollinating reproduction system of red clover implies phenotyping numerous individual plants under water-limited conditions, crossing the better individuals, and continuing selection for multiple cycles [13,14]. Moreover, DR is a quantitatively inherited trait. As a consequence, although some results have been achieved [15], breeding progress for DR is generally slow in red clover. Knowing which genomic regions are associated with a beneficial drought response can be the basis to develop molecular markers for DR in red clover. Molecular markers would allow for the screening of large numbers of young plants and genetically identify promising material at the start of a breeding trial, reducing the need for a laborious phenotypic screening [13]. In addition, candidate genes related to DR could be revealed, which may increase insights into the physiological mechanisms driving drought responses in red clover. Genome-wide association studies (GWAS) are a widely used approach with which to study the genetic basis of phenotypic variation in crops [16]. A prerequisite for GWAS is the availability of genome-wide molecular marker data. In cross-pollinated crops, which include most grassland crops like red clover, phenotypic data from field or greenhouse experiments and genotyping data are more relevant at the population level than at the individual plant level [17]; reduced representation genome sequencing methods, like genotyping-by-sequencing (GBS), are an effective method with which to quantify allelic variants at the population level (genome-wide allele frequency fingerprinting). GWAS have been used to elucidate the genetic control of complex traits in various grassland legumes such as forage quality traits [18] and autumn dormancy [19] in lucerne (*Medicago sativa*), and disease resistance [20] and freezing tolerance [21] in red clover. Nonetheless, our knowledge on the genetic control of drought responses in grassland legumes, and especially in red clover, remains limited.

In previous work [22], we investigated drought responses in 395 red clover accessions of the EUCLEG collection in a two-year field trial with mobile rain-out shelters. Drought caused substantial reductions in canopy cover (CC) and canopy height (CH), with visible reductions starting roughly two weeks after the onset of drought and continuing during the recovery period until late in the growing seasons. Plants reacted differently to drought in the first and second year of production, as plants were older in year 2, and may have developed deeper rooting that could alter drought responses. The same collection of 395 accessions has been genotyped by Frey et al. [20] using a pooled GBS approach followed by single nucleotide polymorphism (SNP) frequency profiling using the Milvus red clover genome assembly [23]. Recently, a new, high-quality, long read-based assembly of the red clover genome of a different red clover genotype (HEN-17) became available [5]. This HEN-17 genome assembly presents a substantially improved reference genome sequence, with improved per-base quality, more than 500 times fewer contigs, and a three-fold increase in contig N50 compared to the previous assembly [5]. The HEN-17 assembly is nearly 20% longer than the Milvus assembly (413.5 Mbp vs. 350 Mbp), which is closer to the predicted genome size of red clover. The Milvus genome annotation contains 39,948 protein coding genes; the HEN-17 genome annotation contains 33,610 protein coding genes.

The present work was performed in the framework of the EUCLEG project that focuses on legume crop breeding. In the present paper, we build further on the following two studies: the study by Frey et al. [20], in which 395 red clover populations with relevance for European breeding activities were genotyped by GBS; and the study by Vleugels et al. [22], in which the phenotypic variation for DR was investigated in the same collection. The objectives of the present study were, as follows: (i) to perform GWAS to identify quantitative trait loci (QTLs) for DR in red clover; and (ii) to identify potential candidate genes in the genomic regions underlying the QTL associated with DR in red clover. We have performed GWAS analysis in parallel based on two SNP sets, using either the Milvus assembly [23], or the HEN-17 assembly [5] as references for SNP calling.

## 2. Materials and Methods

### 2.1. Phenotypic Data for DR

**Figure 1:**
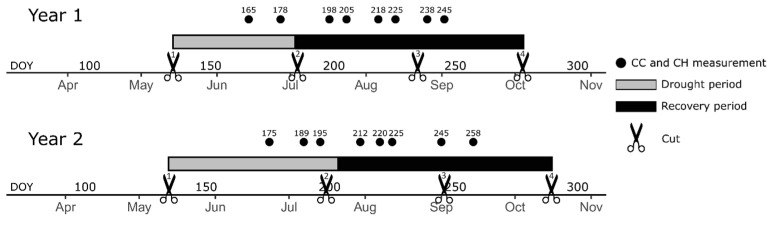
Timeline of the phenotypic data from Vleugels et al. [22] that were used for genome-wide association study (GWAS).

Phenotypic data were used from our previous work [22], in which we phenotyped a panel of 395 red clover accessions for drought responses in two parallel fields, as follows: a ‘drought-treated’ field where drought was simulated using rain-out shelters; and a ‘control field’ that was irrigated and served to calculate relative performance indices. Four cuts were taken per year, and drought periods lasting six to eight weeks were imposed between the first and second cuts in two subsequent production years or growing seasons (Figure 1). CC and CH were monitored throughout the two growing seasons. Subsequently, relative performance indices (Yr), displaying the relative performance of the drought-treated plots compared to the control plots, were obtained for each accession and each trait, using the following formula:

Yr = (Control − Drought)/Control(1)

Positive Yr values for CC or CH indicate lower observed values of coverage or height for that accession in the drought-treated field compared to the control field.

For the present study, we defined drought resilient accessions as accessions that display small differences in terms of CC or CH either during the drought period (cut 1–2) or in two recovery periods (cuts 2–3 and 3–4). We used Yr indices for CC and CH for both growing seasons, year 1 and year 2, from the middle of the drought treatments until the middle of the 2nd growing periods after drought, further named recovery periods. To ensure meaningful Yr indices, only observations obtained at least 10 days after a cut were included. A timeline visualizing the trial and the phenotypic data used in this paper are given in Figure 1. For year 1, we included Yr indices for CC and CH at DOY(day-of-year) 165, 178, 198, 205, 218, 225, 238 and 245; for year 2, Yr indices for CC and CH at DOY 175, 189, 195, 212, 220, 225, 245 and 258 were used. This rendered a total of 32 traits, 16 in year 1 and 16 in year 2 (Table 1). GWAS was performed individually for each Yr index, further named DR trait.

### 2.2. GBS

A full description of the methods for GBS genotyping and SNP calling of the 395 red clover accessions is provided in Frey et al. [20]. We used SNP data obtained with the Milvus genome assembly [23] as a reference for read mapping, as available from Frey et al. [20]. In parallel, we ran the SNP calling analysis for the GBS data of the EUCLEG red clover collection using the more recent HEN-17 genome assembly as reference sequence [5]. Read mapping was performed using Bowtie (v2.4.4) [24], while SNP calling and filtering were performed using *bcftools* (v1.16) [25]. SNP data were filtered for biallelic genotype calls with a minimum read depth of 30 and at least 100 accessions genotyped per SNP position.

### 2.3. GWAS

GWAS was performed using the R-package ‘GAPIT’ in R version 4.2.2., implemented in RStudio [26,27], applying the Bayesian-information and linkage-disequilibrium iteratively nested keyway (BLINK) model [28,29]. BLINK was chosen as the preferred model for its superior handling of genetic diversity and population structure, as well as its ability to minimize redundant signals across linkage blocks. While other models such as the general linear model (GLM) tests markers independently, BLINK offers a more refined and efficient approach by retaining only the most significant SNP from each chromosomal region. This allows for a clearer distinction of significant associations, avoiding noise from multiple linked SNPs within the same region. A key strength of BLINK is its ability to merge marker–trait associations within a linkage block, reducing false positives and enhancing the precision of detected associations. By focusing only on the most relevant SNP in each region, BLINK avoids signal dilution that can occur when multiple linked markers are considered, giving it a distinct advantage over other GWAS models.

For our analysis, BLINK was configured with default settings and a linkage disequilibrium threshold of 0.2, ensuring that SNP–trait associations were both robust and well-controlled. Associations were considered significant if they surpassed a threshold of -Log10 (*p*-value) ≥ 6. To evaluate the contribution of each SNP, the phenotypic variance explained (R^2^) was calculated via linear regression between phenotypic data and SNP genotype data using the *lm()* function in R 4.2.2. SNPs were selected based on an R^2^ threshold of greater than 0.05 and a Minor Allele Frequency (MAF) above 0.05 within the total association mapping panel. Closely linked SNPs were inspected carefully, and if one SNP exhibited lower *p*-value, MAF, or R^2^ values compared to others, the lower-performing SNP was removed. Effect sizes and the phenotypic variance explained by SNPs associated with traits were represented by the regression coefficient (β) and R^2^, derived from a linear model with Best Linear Unbiased Estimations (BLUEs) as the response variable, with SNPs treated as fixed effects.

### 2.4. Identification of Candidate Genes

For the 21 significant SNP–trait associations found in the HEN-17-derived SNP set, flanking genes were identified within a 50 kb region upstream and downstream of the significant SNP in the HEN-17 assembly [5] (GCF_020283565.1). Subsequently, we performed a *BLASTx* search with the corresponding gene sequence against the *M. truncatula* proteome to retrieve the functional gene annotations, using PLAZA5.0 [30]. Similarly, for the significant SNP–trait associations from the Milvus-derived SNP set, we first identified flanking genes in the Milvus assembly (50 kb flanking sequences upstream and downstream, assembly GCA_900079335.1; ensemble annotation v2.1 [31]). Subsequently, we used the corresponding gene sequence to perform a *BLASTx* search against the predicted gene set of the HEN-17 assembly, and the *M. truncatula* proteome, as described above, to retrieve the corresponding functional annotation descriptions. For all significant associations, all known genes in a flanking region of ±50 kb from each SNP, together with their orthologs and functional annotations in *M. truncatula* (if available), are given in Supplementary Data File S1. Given the low degree of linkage disequilibrium in red clover [21], in what follows we only considered candidate genes within regions of ±5 kb upstream or downstream from each significant SNP (Supplementary Data File S2).

## 3. Results

Mapping of the GBS read data on the Milvus genome assembly provided 20,137 SNP markers, polymorphic at MAF ≥ 0.05, spread over the seven red clover chromosomes and multiple unanchored scaffolds. The SNP calling on the HEN-17 assembly provided 59,343 SNP markers spread over the 7 chromosomes, and polymorphic at MAF ≥ 0.05.

**Figure 2:**
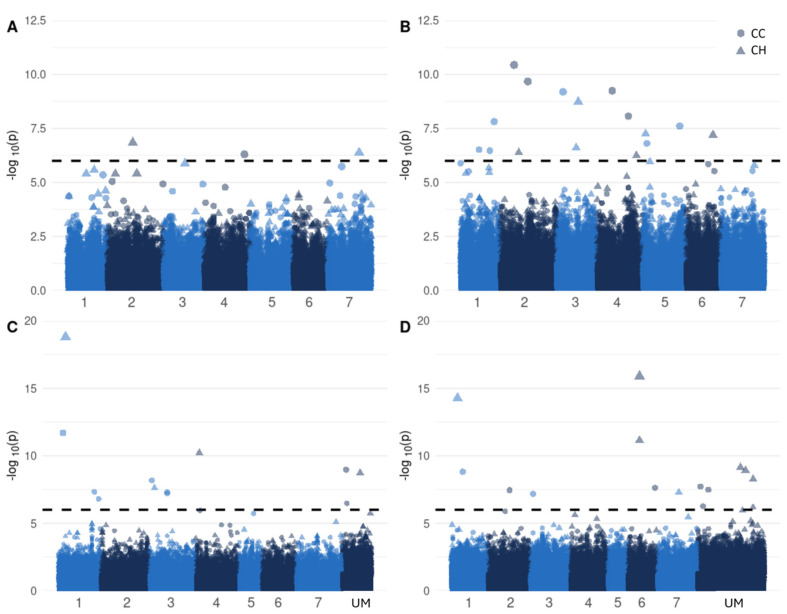
Manhattan plots from GWAS on DR in red clover, displaying significant associations for: (**A**) traits in the drought period; (**B**) the recovery periods of both years using the HEN-17-derived SNP set; (**C**) traits in the drought period; and (**D**) the recovery period of both years using the Milvus-derived SNP set. The dotted line represents the significance threshold after Bonferroni correction (α = 5%). Colors represent the different linkage groups (LG1 to LG7), and unmapped (UM) contigs.

As the recent long read-based HEN-17 assembly [5] presents an improved reference genome sequence, with substantially more genes located on chromosomes instead of on scaffolds compared to the Milvus assembly [23], we first performed GWAS using the HEN-17-derived SNP set. This resulted in 19 SNPs that were significantly associated with improved drought responses after Bonferroni correction (α = 5%), explaining between 5.3 and 23.2% of the phenotypic variation for various DR traits (Figure 2A, Table 2). Three SNPs were associated with drought responses during the drought period, while the remaining 16 SNPs were associated with responses during the recovery periods. The full results of the GWAS are shown in Supplementary Data File S1. The following two SNPs explained more than 15%: NC_060063.1_4412473 on LG5; and NC_060064.1_36266159 on LG6. No candidate genes were identified in the flanking regions (±5 kb) of these SNPs. GWAS indicated additional SNPs with minor effect. First, two genes related to cell wall structuring were highlighted as follows: genes with homology to arabinosyltransferase (year 1, CC_198: 9.2% of variation explained); and pectinase inhibitors (year 2, CC_212: 5.9% of variation explained). Second, associations were found with SNPs near genes encoding sugar-modifying proteins, such as glycosyltransferase genes for CC_195 and CC_225 in year 2, explaining 11.3% and 5.3% of the phenotypic variation, respectively. Additionally, an SNP flanking a gene encoding an α-galactosidase was significantly associated to CC_198 in year 2, explaining 8.4% of the phenotypic variation. Third, an SNP significantly associated to CH_225 in year 1, explaining 5.9% of the phenotypic variation, was located in a gene encoding an ureide permease. Finally, we found associations with an SNP explaining 6.6% of the phenotypic variation for CC_258 in year 2, located in a gene containing an Ma3-binding domain, and an SNP explaining 8.4% of the phenotypic variation for CC_198 in year 1, with its flanking gene encoding a MAP kinase.

As the associations revealed using the HEN-17-derived SNP set explained only minor percentages of phenotypic variation, we decided to re-run our GWAS using the Milvus-derived SNP set. GWAS analysis with the Milvus-derived SNP set yielded 29 SNPs that were significantly associated with DR: 14 SNPs in the drought period and 15 SNPs in the recovery period (Figure 2B, Table 2). Considerably larger percentages of phenotypic variation were explained compared to the HEN-17-derived SNP set, up to 42.3%. Eleven SNPs on LG1, LG3, LG6, LG7 and an unanchored scaffold (LG1_12991269, LG1_24162580, LG1_3190272, LG1_4925076, LG1_4925141, LG3_1774487, LG3_3530352, LG6_9239088, LG6_9239175, LG7_16795407, and scaf_678_58484), among which were two closely linked SNPs on LG1, explained more than 25% of the phenotypic variation for DR traits. The full results of the GWAS analysis are shown in Supplementary Data File S1. Next, we located the closest flanking candidate genes for the Milvus-derived SNPs that were significantly associated to DR traits. First, three associations that explained large percentages of phenotypic variation in year 1 (40.3% for CC_178, 34.4% for CH_218, and 32.9% for CH_225) were found with SNPs located in genes encoding transmembrane proteins. During the drought periods, we found associations with SNPs flanking four candidate genes. The first association was found in year 2 between CH_189 and an SNP in a flowering control gene, explaining 36.6% of the phenotypic variation. Furthermore, CH_178 and CH_189 were associated with two SNPs flanking a DEAD-box ATP-dependent RNA helicase gene (26.5% and 29.1% of the phenotypic variation explained, respectively). Also, an association was found between CC_178 and an SNP flanking a P-loop nucleoside triphosphate hydrolase gene, which explained 31.1% of the phenotypic variation. Finally, two associations were found between CC_189 and CC_195 in year 2 and an SNP in a gene encoding a Myb/SANT-like DNA-binding domain protein, explaining 18.8% and 22.2% of the phenotypic variation, respectively. In the recovery periods, an additional association was observed between CC_218 and an SNP in an ubiquitin–protein ligase gene, which explained 12.6% of the phenotypic variation.

## 4. Discussion

GWAS yielded surprisingly different results depending on the genome assembly that was used for SNP calling. Although the HEN-17 reference genome assembly is more complete than the Milvus genome assembly [5], it resulted in fewer significant associations and SNPs that explained lower percentages of phenotypic variation. Multiple strong associations that were detected in the Milvus-derived SNP set, were not detected in the HEN-17-derived SNP set. One possible explanation may be that red clover is a genetically diverse species [3]; the primary sequence of the genome assemblies of divergent genotypes reflects that diversity. If a major QTL locus is present in a set of accessions but absent in the genotype used for the genome assembly, no reads can map to that locus, and no SNPs are called, even if GBS data from the GWAS panel accessions contains reads derived from that locus. On a more subtle level, if the QTL locus is present but the reference sequence is divergent from the alleles present in the GWAS panel accessions, a mapping bias is created against increasingly more divergent alleles. While this allows for the identification of SNPs, it may cause a shift in observed allele frequencies (divergent alleles are lost from the alternative allele frequency count). As a consequence, the power to detect significant markers for that QTL is reduced or lost entirely. Thus, a particular genome assembly may be fit for read mapping and SNP frequency profiling for certain genotypes or populations, while not for others. The set of accessions used for this study comprised mostly European material [22]. The cultivar ‘Milvus’ originates from Switzerland, whereas ‘HEN-17’ is a North American genotype. Possibly, the majority of accessions in this study may have been genetically closer to the Milvus genotype than to HEN-17, which could explain why the Milvus-derived SNP set yielded more and stronger associations. Such within-species genome diversity has previously been described in pan-genome studies, as well as the consequences of a reference bias for quantitative genetics studies, in rice (*Oryza sativa*) [43,44], barley (*Hordeum vulgare*) [45], maize (*Zea mays*) [46], and in the human genome [47]. Notably, such a bias may only be noticed when multiple reference genomes are used in parallel for SNP calling and in subsequent GWAS analysis, which is not routinely conducted in published GWAS studies.

Briefly, when using the HEN-17 assembly for SNP calling, a number of associated SNPs were found flanking genes that have been suggested to be involved in drought responses. Glycosyltransferases and other genes related to cell wall structuring are known to be involved in drought responses [35,36]. In particular, rice plants with over-expression of certain glycosyltransferases display enhanced drought tolerance at the seedling and at the mature stage [35]. The expression of certain α-galactosidase genes increases tolerance to abiotic stress [33], and to drought stress in particular, for example, in New Zealand spinach (*Tetragonia tetragonioides*) [48]. Ureides accumulate under drought and play important roles in drought responses in legume species [48]. Finally, Ma3-binding domains and MAP kinases have been suggested to be involved in stress metabolism [10,37]. It would be interesting to further study these associations in future research.

In what follows, we focus only on the results obtained with the Milvus genome assembly for SNP calling. We performed GWAS for CC and CH, phenotyped during the drought periods and the recovery periods of two growing seasons. This revealed 10 genomic regions on LG1, LG3, LG6, LG7, and scaf_678 that explained more than 25% of the phenotypic variation for DR per SNP–trait combination at various time points during and after drought (Table 2). These SNPs could be used as molecular markers for DR breeding in red clover, and may help to speed up breeding for DR. We uncovered 10 and 13 associations in the Milvus-derived SNP set, explaining on average 18.4% and 25.5% of the phenotypic variation for CC and CH, respectively. First, we identified an SNP in an ubiquitin–protein ligase gene [39]. Ubiquitin–protein ligases are known to negatively regulate ABA-mediated drought responses by ubiquitinating receptor-like protein kinases [39,40]. Furthermore, we identified transcription factors such as MYB transcription factors. The MYB family represents a large, functionally diverse class of proteins that act as transcription factors [42]. In plants, ABA regulates drought responses following a pathway that involves MYB transcription factors [42]. In addition, we found associations with SNPs in drought-responsive genes such as a DEAD-box ATP-dependent RNA helicase gene, and an SNP flanking a P-loop nucleoside triphosphate hydrolase gene [38,49]. DEAD-box RNA helicases are a large family of genes that play key roles in abiotic stress responses through regulating membrane lipid peroxidation [38]. Their involvement in drought responses has been well documented in species including *Arabidopsis thaliana*, rice, and wheat (*Triticum aestivum*) [38,50]. In addition, we found three associations with SNPs in genes encoding transmembrane proteins. Although only few transmembrane proteins have been functionally characterized, various transmembrane proteins have been shown to be involved in drought responses, e.g., in *A. thaliana*, banana (*Musa* sp.) and tobacco (*Nicotiana tabacum*) [34]. Up to 30% of all eukaryote genes are predicted to encode transmembrane proteins, fulfilling functions in signal transmission, the transport of nutrients, energy conversion, and stress response [34]. Finally, we found an interesting association with a flowering control gene, which may control plant development and/or the timing of maturity. This association may explain why two distinct drought response strategies exist in red clover, ‘drought tolerance’ and ‘drought survival’, which largely coincide with the maturity type [22,41]. The set of accessions screened here comprised both maturity types that may exhibit allelic variations in the flowering control gene alongside their different drought response strategies.

Different associations were found for the different traits (CC and CH) and the different growing seasons, with remarkably little overlap. In year 2, CC_189 and CC_195 were associated with a single SNP in a Myb/SANT-like DNA-binding domain gene. One SNP–trait association appeared in the two growing seasons: CH_178 and CH_189, respectively phenotyped at the end of the drought periods in years 1 and 2, were associated with SNPs located in a DEAD-box ATP-dependent RNA helicase gene. This poor overlap is not entirely unexpected, as we showed in our previous study that CC and CH act largely independent, and that the overall phenotypic response of the screened red clover accessions differed throughout the growing seasons, and between the two growing seasons [22].

Drought responses have also been studied in other grassland legumes. In lucerne, various putative genes related to drought responses were identified, including genes encoding transcription factors, protein receptor-like kinases, DNA-binding domain proteins, and phospholipase-like proteins [51,52,53,54]. In lucerne, DR is linked to the capacity for sustaining photosynthetic activity, optimizing root development, enhancing water use efficiency, regulating osmotic potential, accumulating minerals (such as potassium) or organic solutes like proline, and adjusting carbohydrate metabolism to favor the accumulation of soluble sugars [55]. Further research is required to unravel the specific mechanisms driving DR in red clover. An important remark in this context is that we conducted GWAS on allele frequencies per accession, unveiling the substantial genetic variation across populations in outcrossing species, such as red clover, and eliminating the necessity to sequence thousands of individuals [17]. When sequencing resources are limited, population-level association studies are a valuable and effective approach for identifying key SNPs. However, to validate significant SNPs and thoroughly characterize allelic variation in candidate genes, genotyping individual genotypes is essential [20].

We have identified a number of candidate genes that are associated with drought responses in the whole set of 395 red clover EUCLEG accessions. However, it is likely that many more drought response genes are present in these accessions. In GWAS, associations that are present in a smaller fraction of accessions only, but uncommon in the total set of accessions, are unlikely to be detected. Therefore, GWAS typically will produce false-negative results, rather than false-positive results. Due to the large genetic diversity in the set of accessions screened in this study, we expect that numerous, more rare associations will be present in a limited set of (related) accessions only. To identify such associations, a separate GWAS study should be conducted with the accessions of interest only. While our study suggested a number of candidate genes, no functional validation was conducted. Future research should perform functional validation of the candidate genes suggested in this study, so that the specific mechanisms driving DR in red clover can be further studied.

## 5. Conclusions

The present study applied GWAS to investigate the genetic control of DR in the EUCLEG red clover collection, relevant for European breeding initiatives. The GWAS identified 48 significant SNP–trait associations for DR traits but depended on the red clover genome assembly used for SNP calling. Based on these SNP–trait associations, we proposed a total of 16 candidate genes for DR in red clover. Several genes known to be involved in drought responses, such as a MYB transcription factor and an ubiquitin–protein ligase, were found by our GWAS. Our results also suggest a link between a flowering control gene, possibly related to the maturity type, and drought responses. A next step could be the implementation of molecular markers for DR in practical breeding programs, which would enable breeders to speed up breeding progress. The genomic regions uncovered in this study can be further investigated to validate their potential contribution to genomic-assisted breeding, or to further characterize important genes associated with DR. This would help to uncover the physiological mechanisms driving DR in red clover.

## Figures and Tables

**Table 1:** Relative performance indices (Yr) for CC and CH used for GWAS, with their average and SD over all 395 accessions observed in Vleugels et al. [22].

Period	DOY	Yr (µ ± SD) for		Cut
		CC	CH	
**Year 1**				
Drought period	165	0.19 ± 0.02	0.31 ± 0.09	
	178	0.44 ± 0.11	0.37 ± 0.09	Cut 2
1st recovery period	198	0.30 ± 0.17	0.35 ± 0.07	
	205	0.28 ± 0.12	0.59 ± 0.09	
	218	0.15 ± 0.06	0.35 ± 0.12	
	225	0.22 ± 0.06	0.07 ± 0.16	Cut 3
2nd recovery period	238	0.12 ± 0.12	−0.59 ± 0.09	
	245	0.19 ± 0.09	0.09 ± 0.05	
**Year 2**				
Drought period	175	0.26 ± 0.08	−0.10 ± 0.14	
	189	0.27 ± 0.09	0.33 ± 0.09	
	195	0.14 ± 0.10	0.22 ± 0.10	Cut 2
1st recovery period	212	0.45 ± 0.09	0.27 ± 0.10	
	220	0.29 ± 0.08	0.31 ± 0.10	
	225	0.17 ± 0.08	0.21 ± 0.10	
	245	0.01 ± 0.03	−0.04 ± 0.11	Cut 3
2nd recovery period	258	0.06 ± 0.11	−2.75 ± 0.24	

DOY: day-of-year; CC: canopy cover; CH: canopy height; Yr: relative performance index; SD: standard deviation. Cuts are indicated with horizontal lines and drought periods are shaded.

**Table 2:** Marker–trait associations from GWAS on DR in red clover, displaying significant associations using the HEN-17-derived and Milvus-derived SNP sets with genes that are known to be involved in drought responses.

Trait	Year	Period	SNP ID	Chr	Pos	Var Expl (%)	Effect (β)	Candidate GeneKnown for DR	Distance (bp)	Protein Annotationin PLAZA 5.0	Reference
Associations using the HEN-17-derived SNP set [5]											
CH_165	19	D	NC_060060.1 34138967	LG2	34,138,967	9.1	−0.14				
CH_178	19	D	NC_060065.1 42224330	LG7	42,224,330	5.6	−0.19				
CC_238	19	R	NC_060059.1 25418144	LG1	25,418,144	8.1	−0.34				
CH_238	19	R	NC_060059.1 39822546	LG1	39,822,546	7.1	0.42				
CC_218	19	R	NC_060059.1 45472125	LG1	45,472,125	11.7	−0.13				
CC_245	19	R	NC_060060.1 18680074	LG2	18,680,074	7.2	0.19				
CH_225	19	R	NC_060060.1 24894651	LG2	24,894,651	5.9	−0.27	*ureide permease 1-like*	SNP in gene	ureide permease-like protein	[32]
CC_205	19	R	NC_060060.1 37047857	LG2	37,047,857	6.0	0.25				
CC_198	19	R	NC_060061.1 9267342	LG3	9,267,342	8.4	0.33	uncharacterized LOC123917029	+1664	α-galactosidase	[33]
								*protein-tyrosine-phosphatase MKP1-like*	−3852	MAP kinase phosphatase	[10]
CH_205	19	R	NC_060061.1 26803013	LG3	26,803,013	11.4	−0.16				
CH_205	19	R	NC_060061.1 29630157	LG3	29,630,157	6.9	0.25	uncharacterized LOC123915918	−171	transmembrane protein, putative	[34]
CH_238	19	R	NC_060062.1 52642219	LG4	52,642,219	11.0	0.67				
CH_225	19	R	NC_060063.1 4412473	LG5	4,412,473	16.4	0.24				
CC_198	19	R	NC_060063.1 6098087	LG5	6,098,087	9.2	0.26	probable *arabinosyltransferase ARAD1*	SNP in gene	secondary cell wall glycosyltransferase family 47 protein	[35]
CH_238	19	R	NC_060064.1 36266159	LG6	36,266,159	23.2	0.74				
CC_195	20	D	NC_060062.1 53931991	LG4	53,931,991	11.3	−0.18	*N-acetyl-α-D-glucosaminyl L-malate synthase*	+616	glycosyltransferase family 4 protein	[35]
CC_212	20	R	NC_060062.1 20504973	LG4	20,504,973	5.9	0.23	*pectinesterase/pectinesterase inhibitor-like*	SNP in gene	pectinesterase/pectinesterase inhibitor	[36]
CC_225	20	R	NC_060062.1 42084107	LG4	42,084,107	5.3	0.14	probable *glycosyltransferase At5g20260*	−278	glycosyltransferase	[35]
CC_258	20	R	NC_060063.1 49890749	LG5	49,890,749	6.6	0.23	*MA3 domain-containing translation regulatory factor 1-like*	SNP in gene	topoisomerase-like protein	[37]
Associations using the Milvus-derived SNP set [23]											
CC_178	19	D	LG1_3190272	LG1	3,190,272	27.5	−0.46				
CH_178	19	D	LG1_4925141	LG1	4,925,141	26.5	−0.12	*DEAD-box ATP-dependent RNA helicase 41*	+352	DEAD-box ATP-dependent RNA helicase	[38]
CC_178	19	D	LG1_24162580	LG1	24,162,580	31.1	−0.13				
CC_178	19	D	LG3_1774487	LG3	1,774,487	40.3	−0.11	uncharacterized LOC123918397	SNP in gene	transmembrane protein, putative	[34]
CH_178	19	D	LG3_3530352	LG3	3,530,352	31.1	−0.14				
CH_165	19	D	LG4_2357602	LG4	2,357,602	5.8	0.29				
CH_165	19	D	scaf_21186_400	scaf_21186	400	10.7	0.15	no genes on scaffold			
CC_178	19	D	scaf_282_123440	scaf_282	123,440	13.1	0.25				
CC_178	19	D	scaf_569_145336	scaf_569	145,336	14.9	−0.04				
CH_218	19	R	LG1_4970479	LG1	4,970,479	21.9	0.25				
CC_218	19	R	LG1_8876185	LG1	8,876,185	5.9	−0.12				
CC_218	19	R	LG2_16875234	LG2	16,875,234	12.6	0.07	*E3 ubiquitin–protein ligase HOS1*	SNP in gene	E3 ubiquitin–protein ligase HOS1	[39,40]
CH_225	19	R	LG6_9239088	LG6	9,239,088	32.9	−0.25	uncharacterized LOC123889235	SNP in gene	transmembrane protein, putative	[34]
CH_218	19	R	LG6_9239175	LG6	9,239,175	34.4	−0.14	uncharacterized LOC123889235	SNP in gene	transmembrane protein, putative	[34]
CH_238	19	R	LG7_16795407	LG7	16,795,407	42.3	0.75				
CC_205	19	R	scaf_17454_702	scaf_17454	702	12.3	0.13	no genes on scaffold			
CH_225	19	R	scaf_215_44822	scaf_215	44,822	22.4	0.47				
CC_205	19	R	scaf_298_215478	scaf_298	215,478	11.1	−0.08				
CH_238	19	R	scaf_677_20023	scaf_677	20,023	12.7	0.42				
CH_238	19	R	scaf_678_58484	scaf_678	58,484	25.0	0.36				
CH_218	19	R	scaf_802_53097	scaf_802	53,097	10.8	−0.17				
CC_218	19	R	scaf_918_18614	scaf_918	18,614	11.9	−0.10				
CH_189	20	D	LG1_4925076	LG1	4,925,076	29.1	−0.13	*DEAD-box ATP-dependent RNA helicase 41*	+287	DEAD-box ATP-dependent RNA helicase	[38]
CH_189	20	D	LG1_12991269	LG1	12,991,269	36.6	−0.14	*flowering time control protein FPA*	SNP in gene	RNA recognition motif (RRM) containing protein	[41]
CC_189	20	D	LG1_26956262	LG1	26,956,262	8.6	−0.09				
CC_189	20	D	LG3_11998225	LG3	11,998,225	18.8	0.17	uncharacterized LOC123913798	SNP in gene	Myb/SANT-like DNA-binding domain protein	[42]
CC_195	20	D	LG3_11998225	LG3	11,998,225	22.2	0.23	uncharacterized LOC123913798	SNP in gene	Myb/SANT-like DNA-binding domain protein	[42]
CC_220	20	R	LG3_2451723	LG3	2,451,723	5.3	−0.15				
CC_245	20	R	LG6_21196281	LG6	21,196,281	8.7	−0.04				

Trait with day-of-year of observation; Trial year; Period: drought (D) or recovery (R); SNP ID; Chromosome (Chr); Position on chromosome (Pos); phenotypic variance explained by that SNP allele (%); Effect size on the phenotype as regression coefficient (β) of the allele frequency; Gene of interest known to be involved in drought responses flanking the significant SNP in the HEN-17 reference genome [5]; Distance (bp) between the SNP and the gene of interest if the gene is upstream (+) or downstream (−) from the SNP; Annotation, functional description of the closest *M. truncatula* ortholog of the candidate gene, and literature references explaining the function of the genes of interest in the context of drought stress.

## Data Availability

All data generated or analyzed during this study are included in this published article [and its Supplementary Data Files].

## Supplementary Materials

The following supporting information can be downloaded at: https://www.mdpi.com/article/10.3390/genes15101347/s1, Supplementary Data File S1: Known genes in a flanking region of ±50,000 bp from each SNP, together with their orthologs and functional annotations in *M. truncatula* (if available) for all significant associations found using the HEN-17-derived and the Milvus-derived SNP sets; Supplementary Data File S2: Known genes in a flanking region of ±5000 bp from each SNP, together with their orthologs and functional annotations in *M. truncatula* (if available) for all significant associations found using the HEN-17-derived and the Milvus-derived SNP sets.

## Abbreviations

ABA: abscisic acid; BLINK: linkage-disequilibrium iteratively nested keyway; CC: canopy cover; CH: canopy height; DOY: day of the year; DR: drought resilience; GBS: genotyping-by-sequencing; GWAS: genome-wide association study; QTL: quantitative trait locus; SNP: single nucleotide polymorphism; Yr: relative performance index.
