# Supplementary material for: GWAS for Drought Resilience Traits in Red Clover (Trifolium pratense L.)
Source: Genes (Basel). 2024 Oct 21;15(10):1347. doi: 10.3390/genes15101347 (PMC11507065; doi:10.3390/genes15101347)
Supplement: Supplementary file 1 [file genes-15-01347-s001.zip › Table S1.pdf]

**Table S1:** Relative performance indices (Yr) for CC and CH used for GWAS, with their average and SD over all 395 accessions observed in Vleugels *et al.* [22].

| Period | DOY | Yr (μ ± SD) for CC | Yr (μ ± SD) for CH | Cut |
|---|---|---|---|---|
| **Year 1** | | | | |
| Drought period | 165 | 0.19 ± 0.02 | 0.31 ± 0.09 | |
| | 178 | 0.44 ± 0.11 | 0.37 ± 0.09 | Cut 2 |
| 1st recovery period | 198 | 0.30 ± 0.17 | 0.35 ± 0.07 | |
| | 205 | 0.28 ± 0.12 | 0.59 ± 0.09 | |
| | 218 | 0.15 ± 0.06 | 0.35 ± 0.12 | |
| | 225 | 0.22 ± 0.06 | 0.07 ± 0.16 | Cut 3 |
| 2nd recovery period | 238 | 0.12 ± 0.12 | -0.59 ± 0.09 | |
| | 245 | 0.19 ± 0.09 | 0.09 ± 0.05 | |
| **Year 2** | | | | |
| Drought period | 175 | 0.26 ± 0.08 | -0.10 ± 0.14 | |
| | 189 | 0.27 ± 0.09 | 0.33 ± 0.09 | |
| | 195 | 0.14 ± 0.10 | 0.22 ± 0.10 | Cut 2 |
| 1st recovery period | 212 | 0.45 ± 0.09 | 0.27 ± 0.10 | |
| | 220 | 0.29 ± 0.08 | 0.31 ± 0.10 | |
| | 225 | 0.17 ± 0.08 | 0.21 ± 0.10 | |
| | 245 | 0.01 ± 0.03 | -0.04 ± 0.11 | Cut 3 |
| 2nd recovery period | 258 | 0.06 ± 0.11 | -2.75 ± 0.24 | |

DOY: day-of-year; CC: canopy cover; CH: canopy height; Yr: relative performance index; SD: standard deviation. Cuts are indicated with horizontal lines and drought periods are shaded.
