# Supplementary material for: GWAS for Drought Resilience Traits in Red Clover (Trifolium pratense L.)
Source: Genes (Basel). 2024 Oct 21;15(10):1347. doi: 10.3390/genes15101347 (PMC11507065; doi:10.3390/genes15101347)
Supplement: Supplementary file 1 [file genes-15-01347-s001.zip › Table S2.pdf]

Table S2: Marker-trait associations from GWAS on DR in red clover, displaying significant associations using the HEN-17-derived and Milvus-derived SNP sets with genes that are known to be involved in drought responses.

Trait	Year	Period	SNP ID	Chr	Pos	Var expl (%)	Effect (β)	Candidate gene known for DR	Distance (bp)	Protein annotation in PLAZA 5.0	Reference
Associations using the HEN-17-derived SNP set [5]											
CH_165	19	D	NC_060060.1 34138967	LG2	34138967	9,1	-0,14				
CH_178	19	D	NC_060065.1 42224330	LG7	42224330	5,6	-0,19				
CC_238	19	R	NC_060059.1 25418144	LG1	25418144	8,1	-0,34				
CH_238	19	R	NC_060059.1 39822546	LG1	39822546	7,1	0,42				
CC_218	19	R	NC_060059.1 45472125	LG1	45472125	11,7	-0,13				
CC_245	19	R	NC_060060.1 18680074	LG2	18680074	7,2	0,19				
CH_225	19	R	NC_060060.1 24894651	LG2	24894651	5,9	-0,27	ureide permease 1-like	SNP in gene	ureide permease-like protein	[41]
CC_205	19	R	NC_060060.1 37047857	LG2	37047857	6,0	0,25				
CC_198	19	R	NC_060061.1 9267342	LG3	9267342	8,4	0,33	uncharacterized LOC123917029	+1664	alpha-galactosidase	[39]
								protein-tyrosine-phosphatase MKP1-like	-3852	MAP kinase phosphatase	[10]
CH_205	19	R	NC_060061.1 26803013	LG3	26803013	11,4	-0,16				
CH_205	19	R	NC_060061.1 29630157	LG3	29630157	6,9	0,25	uncharacterized LOC123915918	-171	transmembrane protein, putative	[49]
CH_238	19	R	NC_060062.1 52642219	LG4	52642219	11,0	0,67				
CH_225	19	R	NC_060063.1 4412473	LG5	4412473	16,4	0,24				

CC_198	19	R	NC_060063.1 6098087	LG5	6098087	9,2	0,26	probable arabinosyltransferase ARAD1	SNP in gene	secondary cell wall glycosyltransferase family 47 protein	[37]
CH_238	19	R	NC_060064.1 36266159	LG6	36266159	23,2	0,74				
CC_195	20	D	NC_060062.1 53931991	LG4	53931991	11,3	-0,18	N-acetyl-alpha-D-glucosaminyl L-malate synthase	+616	glycosyltransferase family 4 protein	[38]
CC_212	20	R	NC_060062.1 20504973	LG4	20504973	5,9	0,23	pectinesterase/pectinesterase inhibitor-like	SNP in gene	pectinesterase/pectinesterase inhibitor	[37]
CC_225	20	R	NC_060062.1 42084107	LG4	42084107	5,3	0,14	probable glycosyltransferase At5g20260	-278	glycosyltransferase	[38]
CC_258	20	R	NC_060063.1 49890749	LG5	49890749	6,6	0,23	MA3 domain-containing translation regulatory factor 1-like	SNP in gene	topoisomerase-like protein	[42]
Associations using the Milvus-derived SNP set [23]											
CC_178	19	D	LG1_3190272	LG1	3190272	27,5	-0,46				
CH_178	19	D	LG1_4925141	LG1	4925141	26,5	-0,12	DEAD-box ATP-dependent RNA helicase 41	+352	DEAD-box ATP-dependent RNA helicase	[47]
CC_178	19	D	LG1_24162580	LG1	24162580	31,1	-0,13				
CC_178	19	D	LG3_1774487	LG3	1774487	40,3	-0,11	uncharacterized LOC123918397	SNP in gene	transmembrane protein, putative	[49]
CH_178	19	D	LG3_3530352	LG3	3530352	31,1	-0,14				
CH_165	19	D	LG4_2357602	LG4	2357602	5,8	0,29				
CH_165	19	D	scaf_21186_400	scaf_21186	400	10,7	0,15	*no genes on scaffold*			
CC_178	19	D	scaf_282_123440	scaf_282	123440	13,1	0,25				
CC_178	19	D	scaf_569_145336	scaf_569	145336	14,9	-0,04				
CH_218	19	R	LG1_4970479	LG1	4970479	21,9	0,25				
CC_218	19	R	LG1_8876185	LG1	8876185	5,9	-0,12				
CC_218	19	R	LG2_16875234	LG2	16875234	12,6	0,07	E3 ubiquitin-protein ligase HOS1	SNP in gene	E3 ubiquitin-protein ligase HOS1	[43-44]
CH_225	19	R	LG6_9239088	LG6	9239088	32,9	-0,25	uncharacterized LOC123889235	SNP in gene	transmembrane protein, putative	[49]

CH_218	19	R	LG6_9239175	LG6	9239175	34,4	-0,14	uncharacterized LOC123889235	SNP in gene	transmembrane protein, putative	[49]
CH_238	19	R	LG7_16795407	LG7	16795407	42,3	0,75				
CC_205	19	R	scaf_17454_702	scaf_17454	702	12,3	0,13	*no genes on scaffold*			
CH_225	19	R	scaf_215_44822	scaf_215	44822	22,4	0,47				
CC_205	19	R	scaf_298_215478	scaf_298	215478	11,1	-0,08				
CH_238	19	R	scaf_677_20023	scaf_677	20023	12,7	0,42				
CH_238	19	R	scaf_678_58484	scaf_678	58484	25,0	0,36				
CH_218	19	R	scaf_802_53097	scaf_802	53097	10,8	-0,17				
CC_218	19	R	scaf_918_18614	scaf_918	18614	11,9	-0,10				
CH_189	20	D	LG1_4925076	LG1	4925076	29,1	-0,13	DEAD-box ATP-dependent RNA helicase 41	+287	DEAD-box ATP-dependent RNA helicase	[47]
CH_189	20	D	LG1_12991269	LG1	12991269	36,6	-0,14	flowering time control protein FPA	SNP in gene	RNA recognition motif (RRM) containing protein	[50]
CC_189	20	D	LG1_26956262	LG1	26956262	8,6	-0,09				
CC_189	20	D	LG3_11998225	LG3	11998225	18,8	0,17	uncharacterized LOC123913798	SNP in gene	Myb/SANT-like DNA-binding domain protein	[45]
CC_195	20	D	LG3_11998225	LG3	11998225	22,2	0,23	uncharacterized LOC123913798	SNP in gene	Myb/SANT-like DNA-binding domain protein	[45]
CC_220	20	R	LG3_2451723	LG3	2451723	5,3	-0,15				
CC_245	20	R	LG6_21196281	LG6	21196281	8,7	-0,04				

Trait with day-of-year of observation, Trial year, Period: drought (D) or recovery (R), SNP ID, Chromosome (Chr), Position on chromosome (Pos), phenotypic variance explained by that SNP allele (%), Effect size on the phenotype as regression coefficient (β) of the allele frequency, Gene of interest known to be involved in drought responses flanking the significant SNP in the HEN-17 reference genome [5], Distance (bp) between the SNP and the gene of interest if the gene is upstream (+) or downstream (-) from the SNP, Annotation: functional description of the closest *Medicago truncatula* ortholog of the candidate gene, and literature references explaining the function of the genes of interest in the context of drought stress.
